# The Impact of Immediate Initiation of Antiretroviral Therapy on Patients' Healthcare Expenditures: A Stepped-Wedge Randomized Trial in Eswatini

**DOI:** 10.1007/s10461-021-03241-9

**Published:** 2021-04-08

**Authors:** Janina I. Steinert, Shaukat Khan, Emma Mafara, Cebele Wong, Khudzie Mlambo, Anita Hettema, Fiona J. Walsh, Charlotte Lejeune, Sikhathele Mazibuko, Velephi Okello, Osondu Ogbuoji, Jan-Walter De Neve, Sebastian Vollmer, Till Bärnighausen, Pascal Geldsetzer

**Affiliations:** 1 TUM School of Governance, Technical University of Munich, Munich, Germany; 2 Clinton Health Acccess Initiative, Boston, USA; 3 Ministry of Health of the Kingdom of Eswatini, Mbabane, Eswatini; 4 Duke Global Health Institute, Duke University, Durham, USA; 5 Heidelberg Institute of Global Health, University of Heidelberg, Heidelberg, Germany; 6 Chair of Development Economics, University of Göttingen, Göttingen, Germany; 7 Division of Primary Care and Population Health, Department of Medicine, Stanford University, Stanford, CA, USA

**Keywords:** Early ART initiation, Universal test-and-treat, Healthcare expenditures, Stepped-wedge trial

## Abstract

Immediate initiation of antiretroviral therapy (ART) for all people living with HIV has important health benefits but implications for the economic aspects of patients' lives are still largely unknown. This stepped-wedge cluster-randomized controlled trial aimed to determine the causal impact of immediate ART initiation on patients’ healthcare expenditures in Eswatini. Fourteen healthcare facilities were randomly assigned to transition at one of seven time points from the standard of care (ART eligibility below a CD4 count threshold) to the immediate ART for all intervention (EAAA). 2261 patients living with HIV were interviewed over the study period to capture their past-year out-of-pocket healthcare expenditures. In mixed-effects regression models, we found a 49% decrease (RR 0.51, 95% CI 0.36, 0.72, p < 0.001) in past-year total healthcare expenditures in the EAAA group compared to the standard of care, and a 98% (RR 0.02, 95% CI 0.00, 0.02, p < 0.001) decrease in spending on private and traditional healthcare. Despite a higher frequency of HIV care visits for newly initiated ART patients, immediate ART initiation appears to have lowered patients’ healthcare expenditures because they sought less care from alternative healthcare providers. This study adds an important economic argument to the World Health Organization’s recommendation to abolish CD4-count-based eligibility thresholds for ART.

**Supplementary Information:**

The online version contains supplementary material available at 10.1007/s10461-021-03241-9.

## Resumen

El inicio inmediato de la terapia antirretroviral (TAR) para todas las personas que viven con VIH tiene importantes beneficios para la salud, pero aún se desconocen las implicaciones en el aspecto económico. Este ensayo controlado aleatorizado por clústers (CRT por sus siglas en inglés) por grupos en distintas etapas pretende determinar el impacto del inicio inmediato de la TAR en los gastos sanitarios de los pacientes en Eswatini. Catorce centros sanitarios fueron asignados aleatoriamente a la transición en uno de los siete periodos de la asistencia estándar (elegibilidad para la TAR en niveles definidos de recuento de CD4) a la intervención de TAR inmediato para todos (EAAA). Se entrevistó a 2.261 pacientes con VIH a lo largo del estudio para conocer sus gastos sanitarios del año anterior. Según los modelos de regresión de efectos mixtos, se observó un descenso del 49% (RR: 0,51; IC del 95%: 0,36, 0,72; p<0,001) en el gasto sanitario total del año anterior en el grupo de la EAAA, y un descenso del 98% (RR 0,02; IC del 95%: 0,00, 0,02; p<0,001) en el gasto en asistencia sanitaria privada y tradicional. A pesar de una mayor frecuencia de visitas deatención de VIH para los pacientes que recién comenzaron laTAR, la aplicación inmediata de laTAR redujo los gastos sanitarios de los pacientes dado que buscaron menos atención de proveedores de asistencia sanitaria alternativos. Este estudio añade un importante argumento económico a la recomendación de la Organización Mundial de la Salud de abolir las restricciones de elegibilidad para la terapia antirretroviral basados en el recuento de CD4.

## Introduction

Since 2015, the World Health Organization (WHO) consolidated guidelines on the use of antiretroviral drugs endorse immediate ART initiation (or “universal test-and-treat”) for all adults, adolescents, and children living with HIV, irrespective of their CD4 cell count or disease stage [1]. Even though patients’ out-of-pocket healthcare expenditures account for approximately 40% of financing for health systems in sub-Saharan Africa [2], policy discussions on when and how to initiate ART have been dominated by epidemiological considerations and the flat-lining in international HIV/AIDS funding, including substantial declines in the United States President's Emergency Plan for AIDS Relief (PEPFAR) support over the past years [3].

While recent epidemiological evidence points to substantial health benefits associated with immediate ART initiation, [4–6] its impact on economic aspects remains more ambiguous [7, 8]. Despite free and universal access to antiretroviral treatment, patients usually have to bear private healthcare-related costs, including supplemental clinic or hospital fees, expenses for transport to and from the clinic, opportunity costs for time and potential income losses, and costs for food, communication, as well as childcare during longer stays [9, 10]. These out-of-pocket payments can exert considerable financial burden on households living close or below the poverty line and often account for a large proportion of a household’s monthly expenditures [11, 12]. To finance increased healthcare costs, ART patients are frequently forced to sell assets or borrow money, likely at high interest rates [2, 9]. Empirical evidence suggests that these coping strategies may not only have detrimental effects on their economic welfare but can also hamper adherence to ART and retention in care [9, 13–15].

Given this important economic burden on patients living with HIV and that patients’ out-of-pocket healthcare expenditures are a major source of health system financing, [13] the impact of immediate ART initiation on patients’ healthcare expenditures should be a key consideration in policy decisions on when to initiate ART. Thus far, it is unclear whether immediate ART initiation positively or negatively affects healthcare expenditures. On the one hand, immediate ART initiation increases the frequency of ART care visits and may, thus, lead to higher out-of-pocket expenditures. On the other hand, patients on a “test-and-treat” programme may reduce care-seeking from alternative healthcare providers and have a lower risk of HIV-related opportunistic infections and hospitalizations, both potentially reducing healthcare expenditures.

To answer this question, we conducted a stepped-wedge cluster-randomized controlled trial of the “Early Access to ART for All” (EAAA) programme in Eswatini. Eswatini faces the world’s highest HIV prevalence [16]. Women are disproportionately affected at an HIV prevalence of 35% in the age group of 15–49 years, compared to a prevalence of 18% among men in the same age group [17]. Levels of HIV-related stigma are generally high and can hamper linkages to “test-and-treat” programs, especially for clinically asymptomatic people [18, 19]. To our knowledge, this is the first randomized study to date that allows for a causal comparison of patient-borne expenditures before and after introduction of an immediate ART initiation policy. Findings from this study have important implications for the viability of “test-and-treat” programs and the overall impact of immediate ART initiation on people living with HIV and their families.

## Methods

The trial [20] and this specific analysis were pre-registered on ClinicalTrials.gov (NCT02909218 and NCT03789448, respectively).

### Study Setting

The study was carried out in the Hhohho Region of Eswatini (known as “Swaziland” until April 2018), located in the North-Western part of the country. Study sites were 14 government-managed health facilities with varying volumes of patients (see Figure SI and Supplemental Table SI). At the study’s outset, all health facilities were providing comprehensive HIV care and treatment according to the national adult HIV treatment guidelines.

### Trial Design

The study was conducted over the course of three years and used a stepped-wedge randomized trial design across fourteen government-managed health facilities. Prior to randomization, health facilities were grouped into seven pairs according to geographic proximity and catchment size. Six pairs of health facilities were cluster-randomized into one of seven sequences. Each facility pair thus transitioned sequentially from the standard of care (control condition) to "Early Access to ART for All" (EAAA) (treatment condition) over the course of three years, namely from September 2014 to August 2017 (see Table 1). One of the seven pairs was excluded from the randomization process and transitioned to the intervention first to accommodate the research timeline for the study’s accompanying qualitative component. Randomization was performed by the trial statisticians using a random number generator in Excel. No stratification was used. The evaluation consists of data collection over eight time periods, whereby no facility was exposed to the EAAA intervention in the first period while all facilities were exposed in the last period. Investigators, patients, and healthcare providers were blinded to the timing of the transition until 4–6 weeks prior to the EAAA adoption but unblinded to the intervention itself.

**Table 1:** Stepped wedge trial design

Healthcare facility	Sep–Dec 2014	Jan–Apr 2015	May–Aug 2015	Sep–Dec 2015	Jan–Apr 2016	May–Aug 2016	Sep–Oct 2016	Oct 2016–Aug 2017
Mshingishingini nazarene clinic	C	I	I	I	I	I	I	I
Ntfonjeni clinic	C	I	I	I	I	I	I	I
Bulandzeni clinic	C	C	I	I	I	I	I	I
Ndzingeni clinic	C	C	I	I	I	I	I	I
Maguga clinic	C	C	C	I	I	I	I	I
Malandzela clinic	C	C	C	I	I	I	I	I
Pigg's Peak Hospital	C	C	C	C	I	I	I	I
Peak nazarene clinic	C	C	C	C	I	I	I	I
Herefords clinic	C	C	C	C	C	I	I	I
Ndvwabangeni nazarene clinic	C	C	C	C	C	I	I	I
Sigangeni clinic	C	C	C	C	C	C	I	I
Siphocosini clinic	C	C	C	C	C	C	I	I
Horo clinic	C	C	C	C	C	C	C	I
Hhukwini clinic	C	C	C	C	C	C	C	I

*C* control phase, *I* intervention phase

### Control Phase: Standard of Care

During the control phase, patients living with HIV initiated ART in line with the national treatment guidelines effective at the time. HIV care in Eswatini has been decentralized in response to the UNAIDS 90/90/90 target and ART initiation is nurse-led. For the first half of the study period, ART eligibility was restricted to patients with CD4 counts of < 350 cells/mm^3^. From October 2016, eligibility was extended to patients with CD4 counts of < 500 cells/mm^3^.

### Intervention Phase: Early Access to ART for All (EAAA)

During the intervention phase, all patients living with HIV attending the health facility were offered ART, including those with CD4 counts above the thresholds prescribed by national treatment guidelines. Similar to the standard of care, patients were initiated on the recommended first-line treatment regimen unless contraindicated. Participants in the EAAA condition received counselling and ART initiation on the same day and were followedup on via continued monthly counselling after initiation. The counselling strategy was in line with the Ministry of Health’s Integrated HIV Management Guidelines [21] on post-test and adherence guidelines and is summarized in Table SII.

### Data Collection

For each period, data was collected from a random sample of patients living with HIV via standardized paper-based questionnaires in each of the fourteen facilities on randomly selected days. All patients living with HIV (whether on ART or not) who were aged 18 years or older were eligible with the exception of pregnant or breastfeeding women and those unable to give informed written consent due to cognitive impairment. For each facility, data collection days were randomly selected. The timing of data collection visits thus determined whether patients were interviewed during the standard of care phase or after roll-out of the EAAA intervention in their respective clinic. In each facility, patients were approached during their clinic visits and asked to participate in patient exit interviews. The study team was instructed to recruit the next patient entering (rather than exiting) the consultation room so as to avoid an over-representation of patients who spend more time with clinicians [22]. Prior to the interview, patients were informed about the aims and content of the questionnaire and their right to decline or withdraw participation at any point in time. Both verbal and written consent were sought before administering the questionnaire.

### Power Calculations

The sample size for patient exit interviews was determined by funding and feasibility constraints. To determine the statistical power of our analyses (after data collection was completed), we conducted simulation-based power calculations in R using the “SWSamp” package [23]. The simulation approach allows us to account for potential time effects and thus accommodates more flexibility than analytical methods based on the derivation of a design effect or the basic Hussey and Hughes model. Using 1000 simulations and assuming a mean of 250 and a residual variance of 800, our power calculations suggested that with a two-sided test, α = 0.05, eight periods, a mean of 21 participants per healthcare facility per period, and an intra-cluster correlation of ρ = 0.01, we would have 80% power to detect a 54% difference in healthcare expenditures, 60% power to detect a difference of 40%, and 40% power to detect a difference of 34%.

### Outcomes

The primary outcome for this economic evaluation were the total private healthcare expenditures in the 12 months preceding the interview. These were composed of hospitalization co-payments and out-of-pocket expenditures for travel, food, communication, and potential childcare costs, reported for any clinic visit or hospital stay in the past 12 months. Total costs further included past-month expenses for treatment and care associated with the utilization of public sector ART clinics, private clinics, and traditional healers. These costs were summed and multiplied by 12 to compute total past-year healthcare expenditures. Lastly, we captured costs incurred for the clinic visit on the day of the interview, also inquiring whether any income losses occurred due to missing work on the day of the clinic visit. Reported out-of-pocket costs for the current visit were also added to the total past-year count of healthcare expenditures. All expenditures were based on patients’ self-reports and noted down in Lilangeni (SZL). In the results section, expenditure estimates are translated into USD. Conversions are based on the currency exchange rate averaged over the three-year study period from September 2014 to September 2017. The questionnaire is provided in supplementary file VIII.

### Data Analysis

We estimated the intent-to-treat (ITT) effect based on the exposure status of the health facility at the point in time at which the patient was interviewed (see Table 1). Given the skewed distribution of healthcare expenditures, we used a mixed-effects negative binomial regression, with the resulting risk ratios representing the treatment effect. The first model was estimated with a binary indicator (“fixed effect”) for each of the eight time periods in the study to account for time trends and a clinic-level random effect to account for clustering by clinic (Model 1) [24]. In the second model, we relaxed the assumption of a homogenous secular trend across healthcare facilities by including a random slope for period, which allowed the period effects to vary between healthcare facilities (Model 3) [25]. We present results for both models with and without adjusting for participants’ characteristics, including sex and marital status (binary), education (continuous, specifying the number of schooling years), and age (continuous) (Model 2 and 4, respectively) (see Table SIII). In Model 5, we allow for potential non-linearities in education and age by estimating a model with restricted cubic splines. In the supplement, we also present results for a model extension that allows the treatment effect to vary across clusters (see Supplemental Table SIV).

In a final step, we examined a range of alternative explanations for the mechanisms underlying the observed effect of EAAA on the primary outcome. First, we projected expected healthcare expenditures based on the required frequency of ART care visits for (a) pre-ART patients, (b) initiating ART patients, and (c) patients on ART. Subsequently, we determined the observed healthcare expenditures for patients in both study arms based on the self-reported frequency of clinic visits and costs of the pre-ART or ART care visit on the day of the interview. Second, we investigated whether possible changes in a patient’s health status due to EAAA may be responsible for differences in private healthcare expenditures between the study groups by comparing the overall health status between participants interviewed in the control versus the intervention phase. While medical records were not assessed, health status was captured via respondents’ self-reports based on a single question asking about their “overall health during the past two weeks”, with response options ranging from "very good" to "very poor" on a five-point Likert scale. Third, we examined whether EAAA may have led to changes in care-seeking from healthcare providers other than public ART services by analyzing possible differences in usage of private and traditional healthcare services between participants in the control and intervention phase.

## Results

### Sample Characteristics

**Fig. 1:**
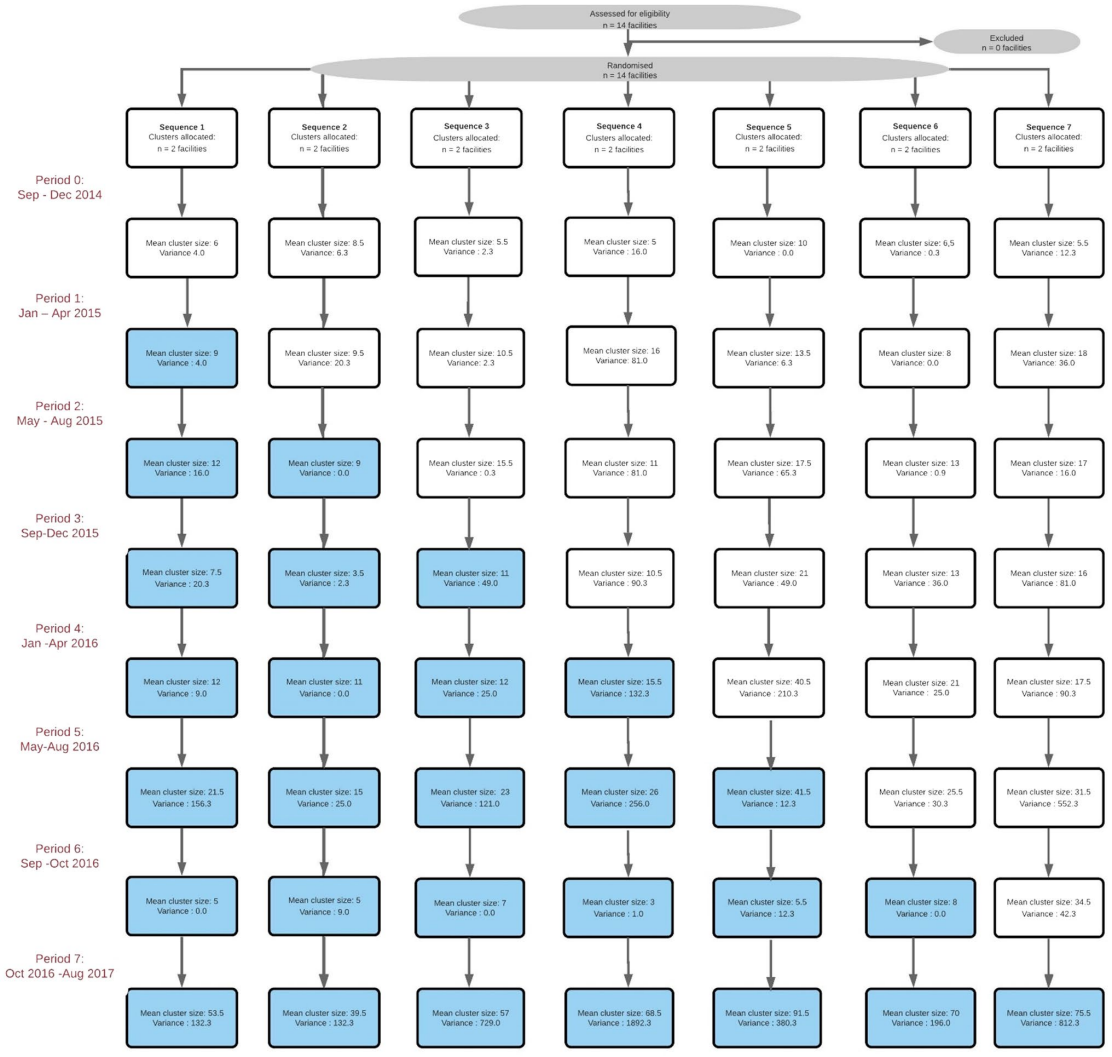
Flow chart of clusters through the trial periods. Intervention (EAAA) period-sequence combinations are indicated in light blue. Each box represents a pair of two facilities that transitioned from the standard of care to the EAAA intervention at the same point in time (Color figure online)

Fourteen healthcare facilities (“clusters”) were enrolled into the trial and 2261 participants were interviewed over the duration of the study. The mean number of participants approached at each time step varied from three participants to 91.5 participants, with a mean of 20.5 participants per facility and sequence (see Fig. 1). This variance is explained by different facility sizes and patient volumes as well as by discrepancies in the duration of individual time steps (see Table 1). Specifically, time step 6 lasted for only one month and ended with the national adoption of a universal test-and-treat approach. The final step (time step 7) spanned eleven months and thus had the highest recruitment rate.

The majority of participants were female (72.1%) and the mean age was 38.4 years (see Table 2). The high share of female participants might be explained by the higher HIV prevalence and a higher utilization of HIV testing and care services among women, such as through antenatal care and family planning services [18, 26, 27] 55.1% were married, only 26.1% of participants were formally employed and almost 20% had not completed any formal schooling.

**Table 2:** Sample characteristics

	Full sample	EAAA intervention	Standard of care
*n*	2261	1406	855
Female, n (%)	1631 (72.1)	998 (71.0)	663 (74.0)
Age, mean (SD)	38.36 (11.93)	38.41 (11.87)	38.28 (12.03)
Age group, n (%)			
18–25 years	261 (11.5)	154 (10.9)	107 (12.5)
26–35 years	846 (37.4)	532 (37.8)	314 (36.7)
36–45 years	562 (24.9)	352 (25.0)	210 (24.6)
45–55 years	354 (15.7)	219 (15.6)	135 (15.8)
> 55 years	238 (10.5)	149 (10.6)	89 (10.4)
Education, n (%)			
No formal schooling	422 (18.7)	268 (19.1)	154 (18.0)
Any primary schooling	839 (37.1)	495 (35.1)	344 (40.2)
Any secondary schooling	989 (43.7)	639 (45.5)	350 (40.9)
Marital status, n (%)			
Married	1242 (55.1)	757 (53.9)	485 (57.1)
Divorced	91 (4.4)	61 (4.3)	30 (3.5)
Widowed	236 (10.5)	153 (10.9)	83 (9.8)
Single, no relationship	564 (25.0)	362 (25.8)	202 (23.8)
Formally employed, n (%)	589 (26.1)	369 (26.2)	220 (25.7)
Any health insurance, n (%)	40 (1.8)	21 (1.5)	19 (2.3)
Months since HIV diagnosis, mean (SD)	59.34 (45.00)	61.01 (45.92)	56.57 (43.31)
Currently on ART, n (%)	2131 (94.8)	1370 (97.9)	761 (89.5)
Months on ART, mean (SD)	45.94 (39.50)	47.08 (39.80)	43.84 (38.86)

*EAAA* early access to ART for all, *SD* standard deviation

**Fig. 2:**
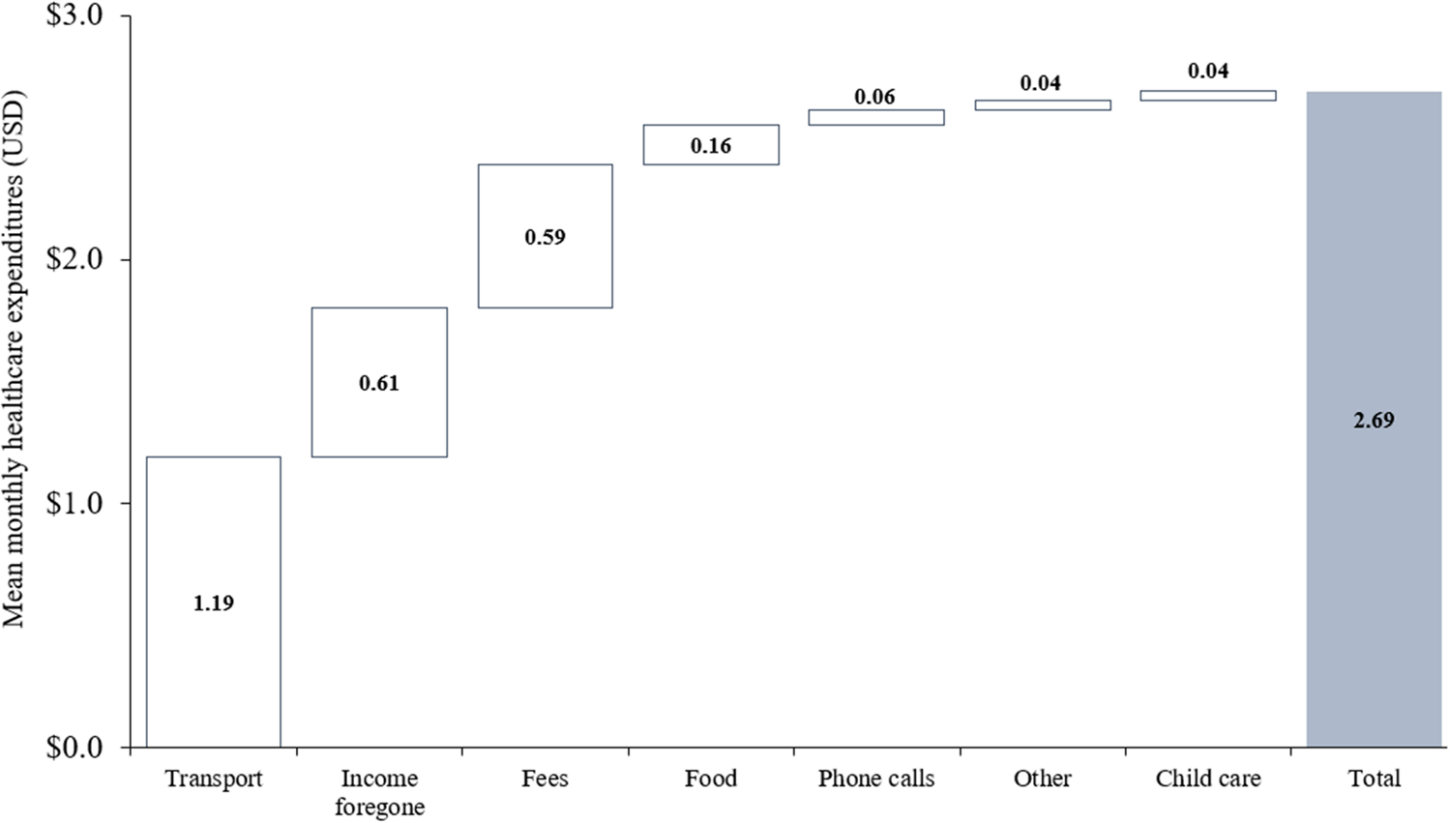
Composition of patients’ monthly healthcare expenditures

The mean and median monthly healthcare expenditure for participants were 2.69 USD (95% CI: 2.34 to 3.04 USD) and 0.83 USD (IQR: 0.38 to 2.12 USD) (equivalent to [18, 26, 27] 35.57 and 11.00 SZL, respectively). Patients’ healthcare expenditures in the full sample (including treatment and control arm) were largely composed of transport costs (44% of total expenditures), followed by the opportunity cost of missing work on the day of the appointment (23% of total expenditures) and fees paid for treatment, counselling, or medicine (22% of total expenditures) (see Fig. 2).

### EAAA Intervention Impact on Healthcare Expenditures

Participants in the EAAA intervention phase reported a 49% decrease (RR 0.51, 95% CI 0.36–0.72, p < 0.001) in their total past-year healthcare expenditures relative to the standard of care group (Table 3). This translates into a reduction in the mean expected healthcare expenditures of 8.73 USD (95% CI − 14.39, − 3.09), or 115.54 SZL (95% CI − 190.30 to − 40.79). This effect size corresponds to Model 4, which includes a random slope for time and adjusts for a number of individual-level covariates. Effect estimates were robust to the different model specifications (Models 1–5) and also remained similar in a model extension allowing the treatment effect to vary across clusters (Supplemental Table SIV). No unintended iatrogenic effects were found.

**Table 3:** Causal effect of the EAAA intervention on total past-year healthcare expenditures

	Model 1^a^		Model 2^b^		Model 3^c^		Model 4^d^		Model 5^e^	
	RR [95% CI]	p value	RR [95% CI]	p value	RR [95% CI]	p value	RR [95% CI]	p value	RR [95% CI]	p value
EAAA Intervention	0.54 [0.39, 0.75]	< 0.001	0.53 [0.38, 0.74]	< 0.001	0.51 [0.36, 0.72]	< 0.001	0.51 [0.36, 0.72]	< 0.001	0.51 [0.36, 0.72]	< 0.001

	Average marginal effect [95% CI]		Average marginal effect [95% CI]		Average marginal effect [95% CI]		Average marginal effect [95% CI]		Average marginal effect [95% CI]	
EAAA Intervention	− 112.70 [− 198.25, − 45.15]		− 119.69 [− 189.99, − 49.37]		− 128.54 [− 204.69, − 52.39]		− 129.94 [− 207.24, − 52.65]		− 129.35 [− 206.29, − 52.41]	
N	2261		2245		2261		2245		2245	

*RR* relative risk, presented for negative binomial regressions, *CI* confidence interval, average marginal effects in SZL

^a^Mixed-effect regression model with random intercept by healthcare facility (cluster) and a fixed effect for study period, thus assuming a homogeneous secular trend across clusters

^b^Same as *Model 1* but with additional control variables,  which were *sex, age, marital status, and education*. All control variables were grand-mean centred. Coefficients for control variables are reported in supplementary Table S1

^c^Mixed-effect regression model with random intercept by healthcare facility (cluster) and a random slope for study period, thus allowing for varying secular trends across clusters

^d^Same as *Model 3* but with additional control variables, including *sex, age, marital status, and education*. Coefficients for control variables are reported in Table S3

^e^Same as *Model 4* but allowing for non-linearities in the control variables age and education through restricted cubic splines with five knots at equally spaced percentiles of the original variable’s marginal distribution

### Why are Healthcare Expenditures Lower in the EAAA Intervention Group?

#### Explanation 1: Impact of the EAAA Intervention on ART Care Visit Frequency

Eswatini’s national treatment guidelines prescribe three-monthly visits for both pre-ART and ART patients and monthly visits for patients initiating ART during the first six months of treatment. If patients were to adhere to the prescribed number of clinic visits, then we would expect patients to incur a mean yearly healthcare cost of 4.23 USD (95% CI 3.84–4.63 USD) versus 3.96 USD (95% CI 3.44–4.47 USD) under EAAA and the standard of care, respectively, based on the distribution of ART-initiating to pre-ART and ART patients in each study phase (Supplemental Table SV). When additionally using the self-reported past-month frequency of ART visit attendance to take into account that some patients miss visits and that this non-attendance varies by patient type (pre-ART, ART-initiating, and ART patients), we estimate that patients incur a mean yearly healthcare expenditure of 3.42 USD (95% CI 3.00–3.84 USD) and 2.84 USD (95% CI 2.50–3.17 USD) under EAAA and the standard of care, respectively. Once all patients living with HIV in EAAA have been on ART for at least 6 months, the corresponding yearly expenditure would only be 3.37 USD (95% CI 2.95–3.78 USD) under EAAA. These projections are thus at odds with out empirically observed results, which reveal higher yearly healthcare costs in the standard of care group.

#### Explanation 2: Impact of the EAAA Intervention on Health Status

We did not find evidence of significant differences in illness levels between both groups (OR 0.95, 95% CI 0.75–1.21, p = 0.690, see Supplemental Table SVI), which could explain differences in healthcare expenditures between study arms.

#### Explanation 3: Impact of the EAAA Intervention on Care-Seeking from Other Healthcare Providers

Patient-borne expenditures on care-seeking from private doctors and traditional/faith healers were significantly lower in the EAAA than in the standard of care group. The relative effect size varied from 94 to 98% lower healthcare expenditures in the EAAA intervention group relative to the standard of care group, depending on the regression model used (Table 4). The incurred patient-borne costs in the private/traditional sector largely consisted of fees charged for consultation, treatment, and care, as well as of other, unspecified expenses, possibly for specific drugs and herbs (see Supplemental Table SVII).

**Table 4:** Causal effect of the EAAA intervention on healthcare expenditures, separately for expenditures in the public versus the private or traditional sector

Healthcare expenditures	Model E^a^		Model F^b^		Model G^c^		Model H^d^	
	RR [95% CI]	p value	RR [95% CI]	p value	RR [95% CI]	p value	RR [95% CI]	p value
Formal public healthcare sector	1.20 [0.93, 1.55]	0.152	1.23 [0.96, 1.59]	0.100	1.18 [0.90, 1.53]	0.230	1.22 [0.95, 1.58]	0.127
Private or traditional sector	0.04 [0.00, 0.37]	0.005	0.03 [0.00, 0.44]	0.010	0.06 [0.04, 0.11]	< 0.001	0.02 [0.00, 0.12]	< 0.001

	Average marginal effect [95% CI]		Average marginal effect [95% CI]		Average marginal effect [95% CI]		Average marginal effect [95% CI]	
Formal public healthcare sector	6.52 [− 1.17, 14.20]		6.33 [− 1.29, 13.96]		4.98 [− 3.17, 13.10]		5.93 [− 1.75,13.62]	
Private or traditional sector	− 49.58 [− 180.15, 80.99]		− 53.37 [− 203.18, 96.43]		− 179.37 [− 692.73, 333.99]		− 125.90 [− 505.09, − 136.10]	
N	2261		2093		2261		2093	

*RR* relative risk, presented for negative binomial regressions, *CI* confidence interval, average marginal effects in SZL.

^a^Mixed-effect regression model with random intercept by healthcare facility (cluster) and a fixed effect for study period, thus assuming a homogeneous secular trend across clusters

^b^Same as *Model E* but with additional control variables, which were *sex, age, marital status, education*. All control variables were grand-mean centred

^c^Mixed-effect regression model with random intercept by healthcare facility (cluster) and a random slope for study period, thus allowing for varying secular trends across clusters

^d^Same as *Model G* but with additional, grand-mean centred control variables, including *sex, age, marital status, education*

## Discussion

This study presents the first causal estimates of the impact of immediate ART for all on the healthcare expenditures of people living with HIV. ART initiation and care requires a relatively high frequency of clinic visits for counselling, monitoring, medication pick-ups, and associated treatment and transport cost [9, 10]. Yet, healthcare-related expenditures were significantly lower under EAAA than in the standard of care, which adds economic support to the WHO’s decision to recommend immediate initiation of ART for all people living with HIV. Our data suggests that an important reason for this finding was that patients with HIV who were not yet eligible for ART under the standard of care were more likely to seek care (and thus incur costs) from private or traditional healthcare services than when they were instead initiated on ART under EAAA.

Our findings closely match the results from a cross-sectional study in KwaZulu-Natal, showing that patients living with HIV spent significantly more private resources on traditional and alternative healthcare services if they were not yet enrolled in public sector care [9]. Corroborating this, a qualitative study in the Mpumalanga Province in South Africa revealed high degrees of medical pluralism and frequent “switching” between private, traditional, and public sector healthcare providers in the general population, but little pluralism among patients who have been initiated on ART [28]. These observations point to a possible substitution effect, according to which traditional healers or private doctors are visited more frequently when HIV treatment and care in the public sector is not (yet) accessible. Hence, we may assume that patients who are newly diagnosed with HIV are eager to control and treat their disease, even if their overall health status, at this stage, has not deteriorated significantly. If they are denied immediate ART in a public ART clinic, they may instead resort to the non-public sector for alternative treatments, support, counselling, or receipt of antiretroviral drugs in private clinics [29].

Across sub-Saharan Africa, the density of public-sector healthcare facilities and their staffing levels are often insufficient, and access to public-sector healthcare is thus constrained. In consequence, traditional healers and private physicians may be perceived as more accessible [30]. Private physicians may be appreciated for shorter waiting times, less stigmatization, and higher perceived quality of care [31, 32]. Traditional healers may provide important psychological comfort and relief, and offer care that is sensitive to local cultural beliefs and customs [28, 30, 33]. However, recent studies have also suggested that medical pluralism may have the potential to delay ART initiation and hamper adherence [9, 34, 35]. Accordingly, patients consulting multiple healthcare workers in both the formal and alternative care sector may receive conflicting advice and messages, which can fuel mistrust or alienate them from the healthcare system altogether [28]. Therefore, concerted action by formal and informal healthcare providers is an important prerequisite for preventing patient losses along the HIV care cascade [28, 30, 36, 37].

Indicator 3.8.2 of the sustainable development goals (SDG) declares financial risk protection as one of the key dimensions of universal health coverage. Out-of-pocket healthcare expenditures are considered as catastrophic according to two alternative SDG thresholds: 10% and 25% of total household consumption [38]. Using household consumption data that was collected from a sub-sample of 65.5% of our study participants, out-of-pocket healthcare expenditures reached catastrophic level for 1.8% in the EAAA group and 3.3% in the standard of care group according to the 10% threshold, and 0.5% in the EAAA group and 1.5% in the standard of care group according to the 25% threshold. Policy efforts to provide financial support and more comprehensive insurance coverage may be urgently needed to help people living with HIV cope with catastrophic healthcare expenditures. In line with previous research, we found that the largest share of overall healthcare expenditures was spent on transport.^9^ Promising intervention strategies to alleviate this cost burden could include medication delivery services through community healthcare workers [39], mobile health technologies to promote patient self-management [40, 41] as well as transport subsidies.

While this paper focused on the effect of EAAA on patients’ health expenditures, universal test-and-treat programs may have substantial welfare benefits in addition to reducing health expenditures. As reported in separate manuscripts [42, 43] we did not find that the EAAA intervention improved patient satisfaction nor increased patients’ productivity levels, employment, and living standards. These null effects, however, may be explained by a relatively short follow-up period. In contrast, other studies document a broad range of positive social impacts, including higher disclosure rates [44, 45] higher levels of social support [44–46] and improved quality of life from immediate access to ART [47]. In the longer term, increased social support and life satisfaction could translate into economic benefits, for instance by increasing levels of employment and productivity and thus economic welfare more generally [48, 49].

This study has several limitations. First, this study was only able to assess the impact of EAAA on patients’ healthcare expenditures over a relatively short time horizon. Second, all pre-ART patients were initiated on ART as a facility transitioned to EAAA. The distribution of ART-initiating to more “established” ART patients is, therefore, likely higher in this study than it would be after several years of EAAA implementation. Given that ART-initiating patients had higher healthcare expenditures than did those who were on ART for at least six months, the observed absolute expenditure level in the EAAA arm in this analysis is likely higher than expenditures would be after several years of EAAA implementation. It is, thus, likely that the difference between expenditures under EAAA compared to the standard of care would have been higher if our study had continued for a longer time. Third, other than patients’ self-reported health status, the study did not collect any medical data such as CD4 count levels or viral loads in this sample, largely for reasons of confidentiality. It was therefore not possible to conduct heterogeneity analyses to test whether healthcare expenditures were particularly high among standard of care group participants with potential opportunistic infections and CD4 counts close to (but above) the treatment cutoffs. Fourth, since our recruitment strategy excluded pregnant and breastfeeding women, we were unable to assess whether early access to ART reduced mother-to-child HIV transmission and the economic benefits to women and their households of averting vertical transmission. However, the rate of vertical HIV transmission in a national sample of 18–24-month-old children in Eswatini was below 5% [50] suggesting that such benefits of the EAAA intervention may only be marginal. Fifth, we relied on self-reported expenditure data, which may be prone to possible measurement errors due to recall and reporting biases that are inherent to any money metric measurement [51, 52]. Our expenditure data on hospital stays and associated costs within the last 12 months might be particularly prone to such recall bias. While this issue may have biased our absolute expenditure estimates for the EAAA and standard of care phase, there is no obvious reason why recall or reporting bias should have been different between study phases and thus the comparison between arms is unlikely to be affected. Lastly, the analysis presented in this paper remains incomplete in that it does not capture the potential savings in (private) healthcare spending for averted infections as a result of a “treatment as prevention” programme.

Immediate ART initiation for all patients living with HIV has the potential to reduce HIV incidence and decrease HIV-related mortality and morbidity. This study adds important new evidence to this body of literature by revealing additional economic benefits. Specifically, immediate ART for all in Eswatini decreased patients’ healthcare expenditures, most likely by reducing care-seeking from traditional and private healthcare providers. Along with recent encouraging findings on the benefit for HIV incidence [53], this study thus provides further support to the WHO’s recommendation that countries ought to eliminate eligibility thresholds for ART.

## Supplementary Information

Below is the link to the electronic supplementary material.

Supplementary file1 (DOCX 570 kb)

## Data Availability

The dataset is accessible via: https://osf.io/96w3n/**.**

## Code Availability

The analysis code is accessible via: https://osf.io/96w3n/.
