# Supplementary material for: The Impact of Immediate Initiation of Antiretroviral Therapy on Patients' Healthcare Expenditures: A Stepped-Wedge Randomized Trial in Eswatini
Source: AIDS Behav. 2021 Apr 8;25(10):3194–205. doi: 10.1007/s10461-021-03241-9 (PMC8416844; doi:10.1007/s10461-021-03241-9)
Supplement: Supplementary file 1 — Supplementary file1 (DOCX 570 kb) [file 10461_2021_3241_MOESM1_ESM.docx]

**Supplemental Digital Content**

**Figure S1.** Map of health facilities in HHohho, Eswatini


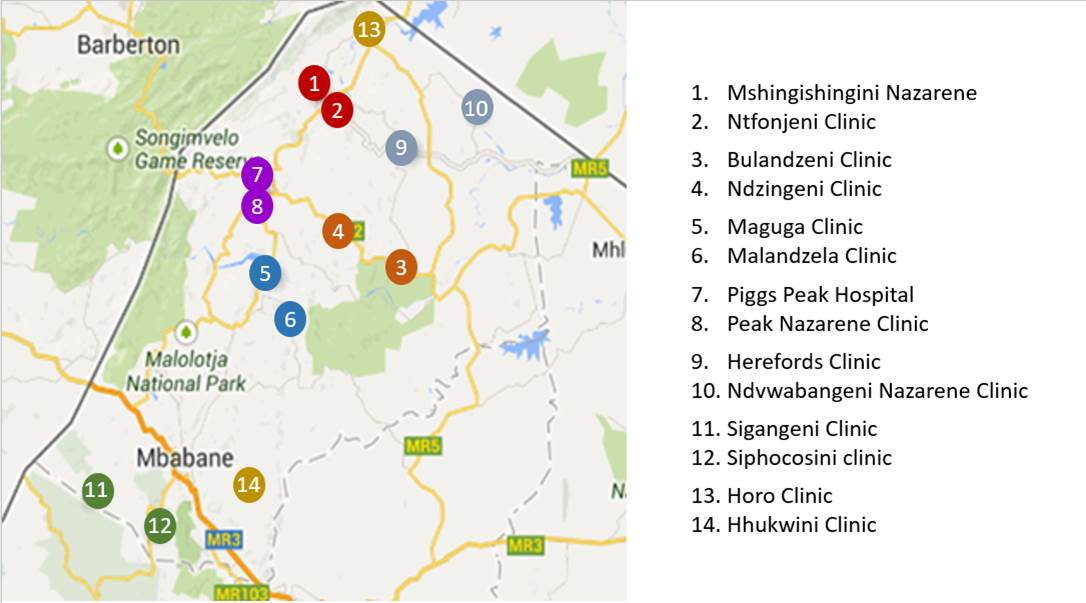


**Table SI.** Clinic characteristics

| **Health Facility Name** | **Facility Type** | **Estimated Catchment Size** | **Number of nurses** | **Number of physicians** | **Number of counsellors** |
| --- | --- | --- | --- | --- | --- |
| Bulandzeni Clinic | Clinic without Maternity | 7,367 | 30 | 0 | 5 |
| Herefords Clinic | Clinic without Maternity | 6,203 | 51 | 0 | 12 |
| Horo Clinic | Clinic without Maternity | 14,317 | 65 | 0 | 13 |
| Maguga Clinic | Clinic without Maternity | 7,996 | 47 | 0 | 16 |
| Malandzela Nazarene Clinic | Clinic without Maternity | 6,690 | 34 | 0 | 2 |
| Mshingishingini Nazarene Clinic | Clinic without Maternity | 6,956 | 47 | 0 | 1 |
| Ndvwabangeni Nazarene Clinic | Clinic with Maternity | 14,868 | 83 | 0 | 3 |
| Ntfonjeni Clinic | Clinic without Maternity | 12,875 | 38 | 0 | 18 |
| Peak Nazarene Clinic | Clinic without Maternity | 5,326 | 41 | 0 | 1 |
| Pigg's Peak Government Hospital | Regional Hospital | 5,417 | 56 | 17 | 7 |
| Sigangeni Clinic | Clinic without Maternity | 6,956 | 47 | 0 | 16 |
| Siphocosini Clinic | Clinic without Maternity | 9,666 | 74 | 2 | 22 |
| Hhukwini Clinic | Clinic without Maternity | 7,658 | 34 | 0 | 2 |
| Ndzingeni Nazarene Clinic | Clinic without Maternity | 7,445 | 20 | 0 | 3 |

**Table SII. Post-test Counselling**

| **Post-test counselling for positive and negative results:** | |
| --- | --- |
| - Simple and clear communication of test results - Client understanding of the result - The opportunity for the client to ask questions - Review of risk reduction plan including condom and lubricant use skills building - Development of a coping strategy for the client - Assessment of referral needs for other services - Discussion of disclosure of test results - Discussion of partner and family referral for HIV testing where appropriate - Clarify misconceptions and myths about HIV transmission and risks | |
| **Additional counselling for negative patients:** | **Additional counselling for positive patients:** |
| - Explanation of window period and recommendation of when to re-test - Discussion of methods to prevent getting HIV such as VMMC, correct and consistent use of condoms, partner reduction etc. - Scheduling of on-going supportive counselling sessions - Referral for prevention services | - Supporting the emotions arising from test result - Discussion of any immediate concerns - Informing individual of available pre-ART and ART services - Information on preventing HIV transmission - Discussion of “positive living” - Scheduling a follow-up counselling session within two weeks to assess coping strategies - Referral to HIV care services and support group |
| **ART adherence counselling:** | |
| - Adopt a “no blame” approach to facilitate open and honest discussion - Actively involve the patient in the decision making of their care and treatment - Use open-ended questions to encourage the client to open up - Perform a subjective assessment of adherence (patient self-reporting) - Perform an objective assessment of adherence to ARVs and OI prophylaxis - Discuss side effects - Work with client to use appropriate reminders (e.g. alarm on cellphone) - Discuss the importance of keeping scheduled clinical and laboratory appointments - Practice confidentiality and reassure clients that their HIV status or anything discussed in the sessions will not be disclosed without their consent - Interventions to support adherence should be individualized to address specific barriers:   - Identify and address any concerns about personal need for ART   - Identify and address specific concerns about taking ART   - Identify and address practical barriers to adherence (limitations in capacity and resources) - Only use interventions to overcome practical problems if there is a specific need. These interventions might include:   - Patients recording their medicine taking (ticking)   - Simplify the dosing regimen   - Use adherence aides like pill boxes and phone alarm reminders   - If side effects are a problem: Consider adjusting the dosage, substitution or other strategies such as changing dose timing or formulation - Patients' knowledge, understanding and concerns about medicines and the benefits they perceive should be reviewed regularly | |

**Source: adapted from** [**Swaziland Integrated HIV Management Guidelines (2015)**](https://hivstar.lshtm.ac.uk/files/2017/11/Swaziland-integrated-HIV-management-guidelines-2015.pdf)

**Table SIII.** EAAA intervention impact with individual-level controls

|  | **Model 2^a^** | | **Model 4^b^** | |
| --- | --- | --- | --- | --- |
|  | RR  [95% CI] | p-value | RR  [95% CI] | p-value |
| ***EAAA***  ***intervention*** | **0.53**  **[0.40-0.74]** | **<0.001** | **0.51**  **[0.36-0.72]** | **<0.001** |
| *Sex* | 0.96  [0.76-1.20] | 0.70 | 0.96  [0.76-1.22] | 0.74 |
| *Age* | 0.98  [0.97-0.99] | <0.001 | 0.98  [0.97-0.99] | <0.001 |
| *Married* | 1.08  [0.89-1.31] | 0.45 | 1.08  [0.89-1.32] | 0.42 |
| *Education* | 1.01  [0.99-1.03] | 0.42 | 1.01  [0.98- 1.03] | 0.55 |
| N | 2245 |  | 2245 |  |

Table Notes: Abbreviations: RR=Relative Risk, presented for negative binomial regressions; CI=Confidence Interval

^a^Mixed-effect regression with random intercept by healthcare facility (cluster) and a fixed effect for study period, thus assuming a homogeneous secular trend across clusters

^b^Mixed-effect regression with random intercept by healthcare facility (cluster) and a random slope for study period, thus allowing for varying secular trends across clusters.

All control variables were grand-mean centered.

**Table SIV** EAAA intervention impact for model extensions

|  | **Model 5^a^** | | **Model 6^b^** | |
| --- | --- | --- | --- | --- |
|  | RR  [95% CI] | p-value | RR  [95% CI] | p-value |
| ***EAAA intervention*** | **0.50**  **[0.32-0.78]** | **0.002** | **0.50**  **[0.32-0.80]** | **0.004** |
| N | 2261 |  | 2245 |  |

Table Notes: Abbreviations: RR=Relative Risk, presented for negative binomial regressions; CI=Confidence Interval

^a^Mixed-effect regression with random interaction between treatment and cluster, thus allowing for treatment effect heterogeneity across clusters

^b^Same as *Model C* but with additional control variables, including *sex, age, marital status, months since HIV diagnosis, months since ART initiation, any past ART*. All control variables were grand-mean centered.

**Table SV.** Patients’ yearly healthcare expenditures depending on ART status: Projections

|  | ***National Treatment Guidelines***  ***[expected projections]*** | | | ***Study Sample***  ***[observed projections]*** | | |
| --- | --- | --- | --- | --- | --- | --- |
|  | Pre-ART | ART initiation | ART patient | Pre-ART | ART initiation | ART patient |
| Average number of visits per year | 4 | 8  (excluding first appointment) | 4 | ***SoC:***  0.78*4  =3.12 | ***SoC:***  0.47*6+0.75*2  =4.32  ***EAAA:***  0.51*6 +0.87*2  =4.80 | ***SoC:***  0.75*4  =3.00  ***EAAA:***  0.87*4  =3.48 |
| Projected patient-borne costs per year *(in SZL)* | Mean:  30.06  Upper bound:  210.00 | Mean:  82.00  Upper bound:  566.32 | Mean:  51.20  Upper bound:  341.84 | ***SoC***:  Mean:  24.15  Upper bound:  163.80 | ***SoC:***  Mean:  44.28  Upper bound:  412.88  ***EAAA:***  Mean:  49.20  Upper Bound:  465.02 | ***SoC:***  Mean:  38.40  Upper bound:  256.38  ***EAAA:***  Mean:  44.54  Upper bound:  297.40 |

*Table notes: Distribution of patient types is pre-ART patients: 10.5% in SoC group; initiating patients: 10.6% in SoC group and 15.6% in EAAA group; patients on ART: 78.9% in SoC group and 84.4% in EAAA group. Based on the reported past-month frequency of health clinic/primary care facility visits in the study sample, we predict the following attendance rates: 78% of pre-ART patients adhered to the recommended number of visits, for ART initiating patients, 47% in the SoC group and 51% in the EAAA group adhered, and for patients on ART, 75% in the SoC group and 87% in the EAAA group adhered. The projected costs per year are based on average patient healthcare expenditures for their current clinic visit excluding the opportunity costs of work missed (depending on patient ART status), i.e. 7.74 SZL (95% CI: 5.61 to 9.87 SZL) for pre-ART, 10.25 SZL (95% CI: 8.51 to 11.99 SZL) for initiating, and 12.80 SZL (95% CI: 11.07 to 14.52 SZL) for ART patients. The upper bound is the average of the top 5% of the expenditure distribution, here 52.50 SZL for pre-ART, 65.90 SZL for initiating, and 85.46 SZL for ART patients.*

|  | **Model A^a^** | | **Model B^b^** | |
| --- | --- | --- | --- | --- |
|  | OR  [95% CI] | p-value | OR  [95% CI] | p-value |
| ***EAAA intervention*** | **0.93**  **[0.73-1.18]** | **0.562** | **0.95**  **[0.75-1.21]** | **0.690** |
| N | 2220 |  | 2220 |  |

**Table SVI.** Causal effect of the EAAA intervention on self-reported poor health

Table Notes: Abbreviations: OR= Odds Ratio, presented for ordinal logit regressions, outcome variable is self-reported overall health, rated from very good to very poor on a 1-5 Likert scale (lower numbers denote better health); CI=Confidence Interval

^a^Mixed-effect regression with random intercept by healthcare facility (cluster) and a fixed effect for study period, thus assuming a homogeneous secular trend across clusters

^b^Mixed-effect regression with random intercept by healthcare facility (cluster) and a random slope for study period, thus allowing for varying secular trends across clusters.

**Table SVII.** Health Care Expenditures by Service Type and Composition of Costs

|  | **EAAA Intervention**  **(N=1406)** | | | | | | **Standard of Care**  **(N=855)** | | | | | | **Difference in means**  **p-value** |
| --- | --- | --- | --- | --- | --- | --- | --- | --- | --- | --- | --- | --- | --- |
| **Costs** | **Total** | *Fees* | *Transport* | *Child Care* | *Food* | *Phone Calls/Texts* | **Total** | Fees | Transport | Child Care | Food | Phone Calls/Texts |  |
| ***Clinic visit***  ***today*** | **11.62**  **(31.83)**  **0-804** | *3%* | *82%* | *4%* | *6%* | *4%* | **13.56**  **(41.15)**  **0-1000** | *8%* | *76%* | *1%* | *10%* | *5%* | 0.67 |
| ***Hospital admissions in past 12 months***  *(6.8% of participants attending in past month)* | **12.17**  **(65.03)**  **0-750** | *40%* | *32%* | *3.5%* | *20%* | *4.5%* | **14.62**  **(116.99)**  **0-3068** | *68%* | *18%* | *0.5%* | *11%* | *2%* | 0.11 |
| ***Past-month clinic visits***  ***(17.7%)*** | **4.13**  **(13.78)**  **0-170** | *15%* | *77%* | *0.5%* | *5%* | *2%* | **3.12**  **(11.03)**  **0-130** | *32%* | *64%* | *0.5%* | *1.5%* | *2%* | 0.96 |
| ***Past-month primary care visits***  ***(5.5%)*** | **2.12**  **(14.51)**  **0-260** | *32%* | *58%* | *0%* | *8%* | *10%* | **4.14**  **(20.73)**  **0-240** | *30%* | *47%* | *1%* | *17%* | *5%* | 0.003 |
| ***Past-month private care visits***  ***(1.0%)*** | **1.61**  **(27.95)**  **0-680** | *74%* | *20%* | *0%* | *6%* | *0%* | **4.72**  **(56.90)**  **0-1270** | *25%* | *8%* | *0%* | *3%* | *5%* | 0.04 |
| ***Past-month traditional care visits***  ***(1.5%)*** | **1.74**  **(28.28)**  **0-770** | *63%* | *29%* | *4%* | *4%* | *0%* | **7.97**  **(75.23)**  **0-1510** | *78%* | *19%* | *0%* | *3%* | *0.2%* | 0.001 |

Table notes: Expenditures in SZL. The table presents the mean, standard deviation in brackets, and the range. The total costs shown here include “any other incurred costs” and can therefore exceed the sum of the individually disaggregated cost items.
